# Identification of Early Salinity Stress-Responsive Proteins in *Dunaliella salina* by isobaric tags for relative and absolute quantitation (iTRAQ)-Based Quantitative Proteomic Analysis

**DOI:** 10.3390/ijms20030599

**Published:** 2019-01-30

**Authors:** Yuan Wang, Yuting Cong, Yonghua Wang, Zihu Guo, Jinrong Yue, Zhenyu Xing, Xiangnan Gao, Xiaojie Chai

**Affiliations:** 1 Key Laboratory of Hydrobiology in Liaoning Province’s Universities, Dalian Ocean University, Dalian 116021, China; wangyuan@dlou.edu.cn; 2 College of fisheries and life science, Dalian Ocean University, Dalian 116021, China; congyuting@dlou.edu.cn (Y.C.); yuejinrong0604@126.com (J.Y.); xxxzy0520@163.com (Z.X.); gao.xn@foxmail.com (X.G.); 3 Bioinformatics Center, College of Life Sciences, Northwest A&F University, Yangling 712100, China; yh_wang@nwsuaf.edu.cn; 4 College of Life Sciences, Northwest University, Xi’an, Shaanxi 710069, China; guozihu2010@yahoo.com

**Keywords:** Salinity stress, *Dunaliella salina*, isobaric tags for relative and absolute quantitation, differentially abundant proteins, proteomics

## Abstract

Salt stress is one of the most serious abiotic factors that inhibit plant growth. *Dunaliella salina* has been recognized as a model organism for stress response research due to its high capacity to tolerate extreme salt stress. A proteomic approach based on isobaric tags for relative and absolute quantitation (iTRAQ) was used to analyze the proteome of *D. salina* during early response to salt stress and identify the differentially abundant proteins (DAPs). A total of 141 DAPs were identified in salt-treated samples, including 75 upregulated and 66 downregulated DAPs after 3 and 24 h of salt stress. DAPs were annotated and classified into gene ontology functional groups. The Kyoto Encyclopedia of Genes and Genomes pathway analysis linked DAPs to tricarboxylic acid cycle, photosynthesis and oxidative phosphorylation. Using search tool for the retrieval of interacting genes (STRING) software, regulatory protein–protein interaction (PPI) networks of the DAPs containing 33 and 52 nodes were built at each time point, which showed that photosynthesis and ATP synthesis were crucial for the modulation of early salinity-responsive pathways. The corresponding transcript levels of five DAPs were quantified by quantitative real-time polymerase chain reaction (qRT-PCR). These results presented an overview of the systematic molecular response to salt stress. This study revealed a complex regulatory mechanism of early salt tolerance in *D. salina* and potentially contributes to developing strategies to improve stress resilience.

## 1. Introduction

Salinity stress greatly affects plant growth and productivity, resulting in cellular energy depletion, redox imbalances, and oxidative damage [1,2]. Scientists have long sought to understand the mechanisms of salt tolerance in plants in order to improve the yield of economically important crops. The unicellular eukaryotic green alga *Dunaliella salina* has extreme salt tolerance, with a unique ability to adapt and grow in salt concentrations ranging from 0.05 to 5.5 M. This organism is an established model for studying plant adaptation to high salinity [3,4], and also has significant pharmaceutical and industrial value, mainly as food for marine aquacultures [5].

To cope with high salinity in its sessile existence, *D. Salina* has evolved a considerable degree of developmental plasticity, including adaptation via cascades of molecular networks [6], which regulate cellular homeostasis and promote survival [1,2,6]. For example, the mitogen-activated protein kinase (MAPK) pathway plays a key regulatory role in plant development as well as in numerous stress responses [2]. We previously cloned the MAPK kinase (MAPKK) cDNA from *D. salina*, and found that its expression was induced upon salt stress [7]. Although salt tolerance in *D. salina* has been studied extensively at the phenotypic, physiological, and genetic level, and many candidate genes associated with energy metabolism, signal transduction, transcription, protein biosynthesis and degradation have been identified [6], the underlying mechanisms remain unclear. Genes responsive to salt stress are regulated at the transcriptional, translational, and post-translational levels. Analysis of the proteome during stress response provides deeper insights into the molecular phenotype since unlike mRNA transcripts, the proteome reflects the actual response of the organism to environmental change [8,9,10,11]. It is necessary to identify the salt stress response proteins to further elucidate the salt tolerance mechanisms in *D. salina*.

Proteomics is a high-throughput approach to study the dynamic protein profile of a cell or organism, and therefore also the intricate molecular networks [2]. Plant cell responses to salinity depend on the tissue, and the severity and duration of the stress, which lead to various changes at the proteome level [12,13]. Recently, salt stress-induced changes have been reported in the protein profiles of rice roots and leaves [9], *Arabidopsis thaliana* roots [11], soybean leaves [13,14], hypocotyls and roots [14] and chloroplasts of diploid and tetraploid black locust [15]. Proteome analysis of *D. salina* subjected to salinity by 2-D gels [16,17] and blue native gels using plasma membrane [3] have shown changes in photosynthesis, protein and ATP biosynthesis, and signal transduction proteins in response to salt stress. Quantitative proteomics and phosphoproteomics have also been applied to explore palmella formation mechanism in *D. salina* under salt stress [4], and have identified several proteins and phosphoproteins as potential candidates for augmenting salt tolerance in this organism. However, previous studies focused on relatively late response to salinity, and did not clarify the early response to short-term salt stress in *D. salina*. The initial phases of stress response in organisms could reveal more profound differences at the proteomic level, as compared to later phases, which could help elucidate novel mechanisms of homeostasis between plant and environment [9,13,18,19]. Therefore, the main objectives of this study were to investigate the early salt stress-response proteome and identify the differentially abundant proteins under short-term salt stress in *D. salina*.

Isobaric tags for relative and absolute quantitation (iTRAQ) is currently one of the most robust techniques used for proteomic quantitation [13,20]. The iTRAQ approach has been used to compare salt tolerant and susceptible cultivars, and has helped identify many proteins that can potentially enhance salt resistance in plants [13,15,20,21]. In the present study, iTRAQ was used to assess proteomic changes and identify differentially abundant proteins (DAPs) in *D. salina* at the early stage of salt stress. NaCl was used in the culture medium to imitate environmental salt stress. We identified 65 and 102 proteins with significantly altered abundance after 3 and 24 h of salt stress respectively; these were then classified into gene ontology (GO) functional groups and pathways. This study advanced our understanding of early salt-responsive mechanisms in *D. salina*. Since *D. salina* is a unicellular organism, the findings reveal how cell can adapt to extreme salinity and provide potential molecular elements for enhancing salinity tolerance in crop plants.

## 2. Results

### 2.1. Identification of *Dunaliella Salina* Differentially Abundant Proteins (DAPs) Using Isobaric Tags for Relative and Absolute Quantitation (iTRAQ)

**Figure 1:**
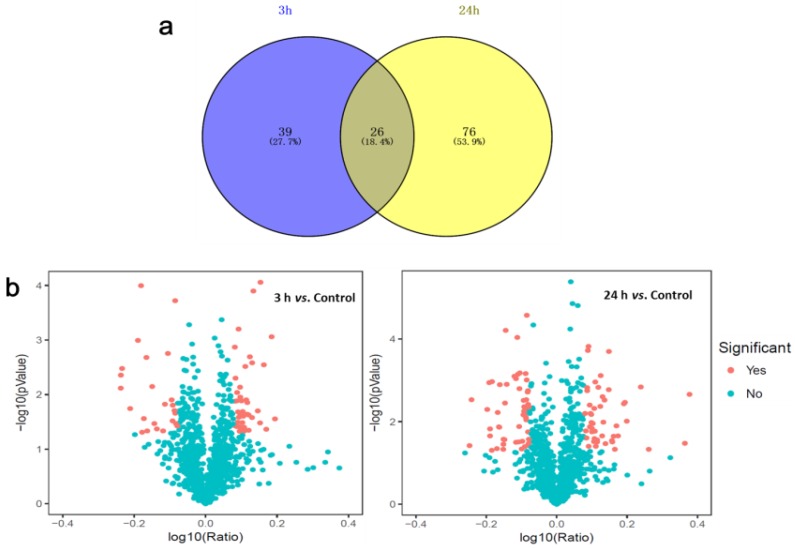
Differentially abundant proteins (DAPs) in response to salt stress in *Dunaliella salina*. (**a**) Venn diagram of 3 and 24 h specific DAPs with overlapping regions indicating the number of common proteins. (**b**) Volcano plots of the proteins quantified during iTRAQ analysis comparing control to 3 and 24 h salt treatments. Each point represents the difference in expression (fold-change) between the two groups plotted against the level of statistical significance. Proteins represented by a filled red square are those with expression that differs at a statistically significant level.

The optimal in-vitro salt stress stimulus with which to explore early responses to salt stress was determined to be 3-M NaCl [3,4] for 3 or 24 h. Proteomes of *D. salina* subjected to the above conditions were analyzed using iTRAQ and liquid chromatography-tandem mass spectrometry/ mass spectrometry (LC-MS/MS). A total of 23,461 spectra were generated from control and salt-treated *D. salina* and were analyzed using the Mascot search engine. 23,461 spectra matched known spectra of 2,283 unique peptides and 1140 proteins from control and salt-treated samples. Detailed annotation information including peptide sequences, accession numbers, matching criteria, unused scores, P-value and sequence coverage of total identified and differential protein species is provided in Supplementary Table S1. The overall changes in protein abundance in *D. salina* after 3 and 24 h salt stress are illustrated in Figure 1. Of the 1140 unique proteins of the control and treated *D. salina*, a total of 141 DAPs were observed in response to salt stress relative to control, with the threshold for upregulated expression >1.2 and downregulated expression <0.83 (*p* < 0.05) [22,23]. 75 of these DAPs were upregulated and 66 were downregulated in the cells after 3 and/or 24 h salt stress (Figure 1a, Supplementary Table S2). Details for each protein are also provided in Table S1. Of these 141 proteins in the control vs. salt treatment, there was an overlap in two categories, which means there were 26 proteins that changed in 3 and 24 h exposures (Figure 1a). Volcano plots show the overall changes to protein abundance in treated compared to control cultures at each time point, with significant difference (*p* < 0.05) in the expression of a few proteins (Figure 1b).

### 2.2. Gene Ontology (GO) Annotation of DAPs in *Dunaliella Salina*

**Figure 2:**
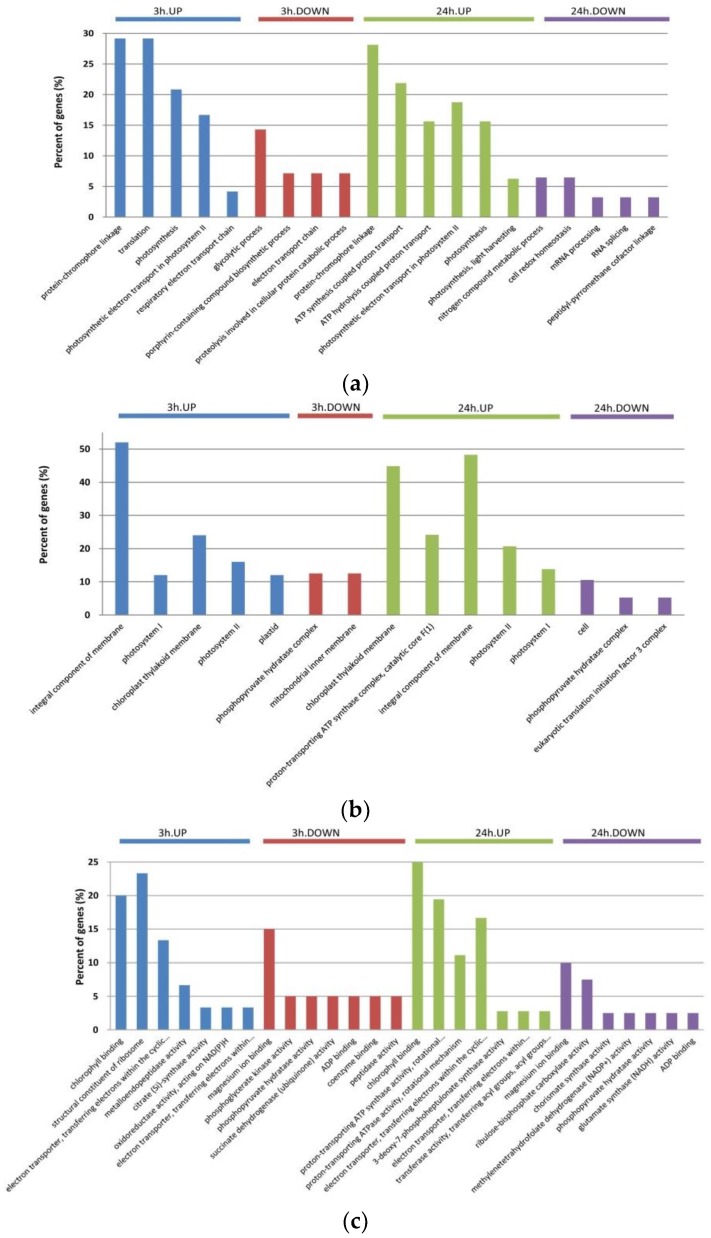
Gene ontology (GO) classification of the DAPs detected at each time point. GO terms in the biological process (**a**), cellular component (**b**), and molecular function (**c**) categories are presented.

To determine the putative functions of DAPs involved in early response to salt stress, they were subjected to GO enrichment analysis. The DAPs were categorized by three sets of ontologies: biological processes (BP), cellular components (CC), and molecular functions (MF) (Figure 2), and a GO term was considered significant at *p*-value < 0.05. As shown in Figure 2a, the significantly enriched GO terms in the BP category included regulation of protein-chromophore linkage, regulation of ATP synthesis coupled proton transport, regulation of ATP hydrolysis coupled proton transport, regulation of translation, regulation of photosynthesis, negative regulation of glycolytic process, negative regulation of nitrogen compound metabolic process, and negative regulation of cell redox homeostasis. In the CC category, integral component of membrane, photosystem I, photosystem II, chloroplast thylakoid membrane and proton-transporting ATP synthase complex were the most significantly enriched terms (Figure 2b). For MF, chlorophyll binding, structural constituent of ribosome, proton-transporting ATP synthase activity, and electron transporter-transferring electrons within the cyclic electron transport pathway of photosynthesis activity were the most significant terms. After 3 h of salt stress, six, seven and four proteins respectively enriched in chlorophyll binding, structural constituent of ribosome, and proton-transporting ATP synthase activity, respectively, were upregulated. After 3 and 24 h of stress, the downregulated DAPs were predominantly related to magnesium ion binding, and after 24 h salt stress the downregulated DAPs were also predominantly related to ribulose−bisphosphate carboxylase activity (Figure 2c).

### 2.3. Kyoto Encyclopedia of Genes and Genomes (KEGG) Analysis of DAPs in *Dunaliella Salina*

**Figure 3:**
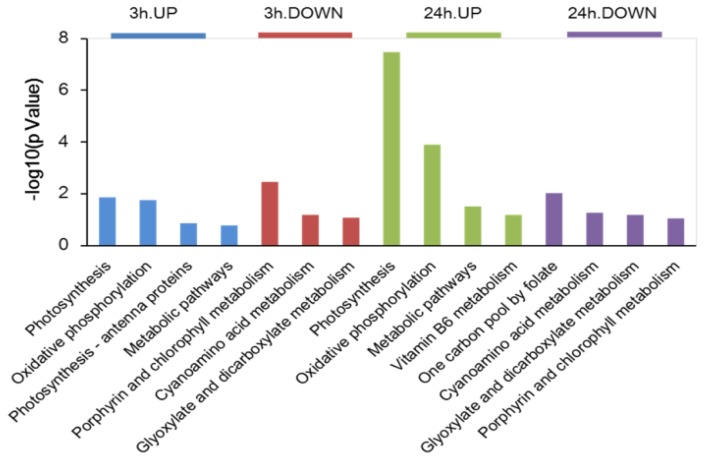
Kyoto Encyclopedia of Genes and Genomes (KEGG) pathway enrichment analysis of the DAPs in *Dunaliella salina* exposed to salt stress at 3 and 24 h. The *x*-axis shows representative enriched KEGG pathways, and the y-axis indicates corresponding p-values of enriched pathways (−log10 *p*-value).

Kyoto Encyclopedia of Genes and Genomes (KEGG) enrichment analysis was used to determine any potential clustering of the DAPs in specific metabolic pathways. Using the KEGG database as a reference, 115 DAPs were annotated and classified into 25 different pathways (Figure 3 and Table S3). After 3 h salt stress, the main KEGG pathway classifications of the DAPs were photosynthesis and oxidative phosphorylation. Porphyrin and chlorophyll metabolism were among the pathways that were most significantly downregulated during the early response. DAPs contributing to photosynthesis, oxidative phosphorylation and metabolic pathways were also significantly enriched after 24 h salt stress, while the ‘one carbon pool by folate’ pathway was significantly downregulated after 24 h. All pathways are listed in Supplementary Table S3.

### 2.4. Search Tool for the Retrieval of Interacting Genes (STRING)-Based Protein-Protein Interaction (PPI) Analysis

**Figure 4:**
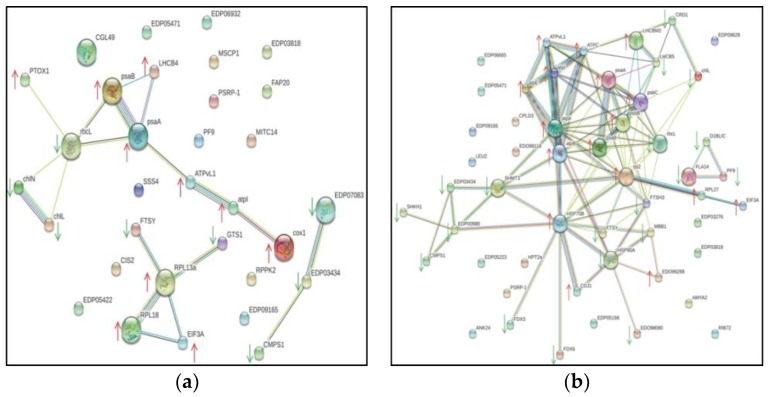
Search tool for the retrieval of interacting genes (STRING)-based protein–protein interaction (PPI) analysis of the DAPs in *Dunaliella salina* exposed to salt stress at each time point. (**a**) 3 h and (**b**) 24 h. The DAPs from *D. salina* were used for constructing PPI network using STRING software. The circles represent proteins while the straight lines represent the interactions between different proteins: gene fusion (red), neighborhood (green), co-occurrence across genomes (blue), protein homology (light green), co-mentioned in PubMed abstracts (yellow), experimentally determined interactions (purple), and interactions determined from curated databases (light blue). The small nodes represent proteins of unknown 3D structure, and large nodes are proteins of known or predicted 3D structure. Red arrows indicate the upregulated DAPs and green arrows indicate the downregulated DAPs.

To determine the regulatory mechanisms of the DAPs and their potential roles in salt stress, we built a regulatory network with the up- and downregulated proteins using STRING analysis; this revealed functional links among the DAPs that were significantly altered after salt stress (Figure 4). Among the 141 DAPs identified by iTRAQ, two regulatory PPI networks of the DAPs containing 33 and 52 nodes (3 and 24 h, respectively) were built. Considerable overlapping was seen among the major clusters, especially with the DAPs involved in photosynthesis, ATP synthesis and ribosome structure regulation pathways. Furthermore, 3 h salt stress induced several protein interactions, including FTSY-RPL13a-RPL18-EIF3A and chlL-chlN-rbcL-psaB-psaA-LHCB4-ATPvL1-atpI-cox1 (Figure 4a). After 24 h salt stress, ATPC, ATPvL1, atpA, atpB, atpE, psaA, psaB, psbB, psbC, HSP70B, rbl2 and EIF3A were the most important protein upregulation hubs, while FTSY, rbcL, HSP90A, LHCB5, EDP00988, SHMT3 and FTSH3 were the most important protein downregulation hubs in the constructed network (Figure 4b). These proteins were not separated and together they formed a related network in response to salt stress.

### 2.5. Analysis of Transcripts Encoding Selected DAPs

**Figure 5:**
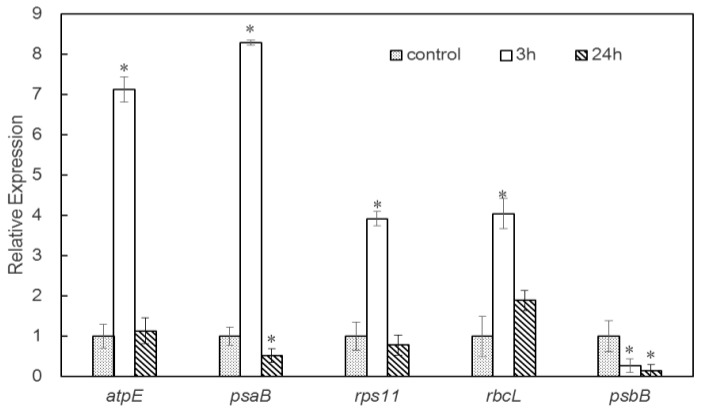
Analysis of transcript levels of the DAPs between salt stress and control conditions by quantitative real-time polymerase chain reaction (qRT-PCR). mRNA expression values were rescaled relative to the control. Statistical significant between experimental and control groups marked with asterisks: (*) *p*-value < 0.05.

To investigate whether the differences in protein abundances were reflected at the mRNA level and to validate the proteomic analysis, the quantitative real-time polymerase chain reaction (qRT-PCR) was used to verify the level of gene expression associated with DAPs between control and salt-treated groups. Four upregulated proteins and one downregulated protein in *D. salina* were selected for verification at the mRNA level. The fold changes of transcript abundances are provided in Figure 5. The transcript levels of four genes displayed the same trend with the abundance of the corresponding protein species, namely, *atpE, psaB, rps11*, and *rbcL*. In contrast, psb*B* showed the opposite trend with the abundance of the corresponding protein in *D. salina* under 3 or 24 h salt stress (Figure 5 and Supplementary Table S2). The discrepancy between the transcription level of the gene and the abundance of the corresponding protein probably resulted from various post-translational modifications [24] under salt stress.

## 3. Discussion

*D. salina* possesses an extraordinary ability to cope with salt stress, and is therefore a model organism for studying salt tolerance [3,4]. In recent years, *D. salina* proteome networks have been analyzed to reveal the molecular basis underlying this organism’s tolerance to different stresses [4,16,25,26]. Previous publications on physiological and molecular changes under salt stress focused majorly on the long-term stress (≥48 h) [3,16,17]. Notably, the response of plants to salinity stress can be determined by the rapid perception of stress shock that occurs within a few hours. The early stress response identified in the previous literature ranged from 1 to 24 h [9,13,18]. However, the adaptive mechanisms underlying *D. salina* in the early response to salt stress (<24 h) at the proteomic level were still unclear. The iTRAQ technology in combination with LC-MS/MS is an effective method for investigating altered protein profiles in plant cells during environmental stress [13,15,20,21]. In the present study, we used an iTRAQ comparative proteomic strategy to analyze the dynamic changes in the protein profiles of *D. salina* exposed to 3 M NaCl [3,4], which simulated environmental salt stress. The aim was to identify protein species to help elucidate the molecular mechanisms associated with early salinity response.

A comparison of the treated and control *D. salina* proteomes revealed 141 DAPs in response to salt stress, indicating a massive metabolic reprogramming. Of these DAPs, 75 were upregulated and 66 were downregulated after both 3 and 24 h of salt stress (Figure 1a). The abundance most DAPs we observed were less than twofold. A possible explanation is that proteins play major roles in most biological processes; as a consequence, protein expression levels are subject to diverse and complex control [27]. Functionally important proteins are subject to higher levels of constraint [28,29]. Functional annotations of these DAPs with GO enrichment analysis showed that most of the upregulated proteins were involved in respiratory metabolism, transport and photosynthesis, while the majority of the downregulated proteins accounted for glycolysis and nitrogen compound metabolic process, in terms of BP. This indicates that salt stress likely affects energy metabolism and ion transportation in *D. salina*, which is useful information for further research into the molecular mechanisms of salt tolerance. DAPs after 3 h of salt stress were predominantly binding proteins that were involved in cellular organization or biogenesis, while the response DAPs after 24 h of salt stress, also primarily binding proteins, were involved in aldehyde and organic acid metabolism. With regards to subcellular location, the significant protein species were mainly enriched in the chloroplast, photosystem, peroxisome, and ribosome (Figure 2). Therefore, our data indicate that *D. salina* is able to mount an early response (as early as 3 h) to salt stress owing to the active stimulation of the critical cell signaling pathways involved in classical stimuli response.

Proteins typically do not exert their functions independently, but rather coordinate with each other in biochemical and physiological processes. Pathway analysis can therefore help reveal cellular processes involved in early salt tolerance [15,20]. These DAPs were further investigated using KEGG database. The main responses to salt stress were seen in proteins that regulate metabolism and energy conversion, carbon fixation in photosynthetic organisms and transport (Figure 3). This is consistent with previous studies which showed that the adaptation of microalgae to stress conditions is accompanied by multiple changes in proteins involved in carbon and energy metabolism [2,9]. In this study, salt stress affected photosynthesis both in 3 and 24 h treated groups. Additionally, we observed an increase in the abundances of many proteins involved in mitochondrial oxidative phosphorylation, indicating an increased need for ATP and energy in response to salt stress.

Photosynthesis in chloroplasts is one of the primary processes that is affected by abiotic stress [30], and the rapid response of photosynthetic machinery and metabolism is pivotal for plants to cope with salt stress [15,30]. We observed that several crucial proteins related to photosynthesis and energy metabolism, such as atpl, ATPvL1, psaA, and psaB, were upregulated upon salt stress in *D. salina*. The ATP synthase alpha subunit (atpA) was upregulated after both 3 and 24 h of salt stress in *D. salina* (Figure 3 and Supplementary Table S2). The increased accumulation of atpA may enhance ATP synthesis to meet increasing energy demands for sustained salt resistance. Previous studies have also documented a regulated expression of multiple subunits of this protein complex under salinity [31,32]. PsaA and PsaB, which encode the large submits of the core complex in PSI that carries the cofactors of the electron transport chain [33], were also upregulated as part of the early response. In plants and green algae, photosystem II and photosystem I consist of a core complex and a light-harvesting complex (LHC) containing electron chain transport cofactors [34]. The LHC protein, together with chlorophyll, captures light energy and delivers it to the photosystems [30]. Under pressure conditions, light harvesting must be reduced to avoid the excessive excitation of and damage to photosystems [34]. We observed that LHCB4 was upregulated after 3 h, while LHCBM2 was upregulated and LHCB5 was downregulated after 24 h salt stress in *D. salina*. The response mechanisms of LHC proteins in early salt stress require further study.

Salt stress can increase the rate of protein unfolding; challenge cellular protein homeostasis for the available folding capacity becomes insufficient. Molecular chaperones or heat shock proteins are a large family of proteins that have the important function of helping other proteins fold and repair misfolding [35,36]. HSP70B and HSP90A play important roles in plant growth and responses to environmental stimuli [37]. In a previous proteomic study, post-translational modifications of HSP90A and HSP90C were speculated to be involved in salt stress responses [38]. In our study, HSP90A was downregulated while HSP70B was upregulated after 24 h salt stress, suggesting an involvement with increased degradation and reduced biosynthesis of proteins during salt stress. This is surprising given that the cooperation between HSP70 and HSP90 systems in chloroplasts has been suggested [39]. Thus, HSP90A may play different roles within cells exposed to salinity.

After 3 h of salt stress, the downregulated metabolic pathways in *D. salina* included not only those of primary but also secondary metabolism. The majority of DAPs related to central pathways, such as porphyrin and chlorophyll metabolism, and one carbon pool related to folate pathways, were downregulated after 24 h salt exposure (Figure 3). These findings indicate that salt stress fundamentally inhibited normal carbohydrate and energy metabolism in *D. salina* during the early stages of response. Both FTSY (a signal recognition particle docking protein) and rbcL (a Rubisco large subunit protein) were arrested in *D. salina* upon salt stress (Supplementary Table S2). This contradicts another study which showed upregulation of FTSY and rbcL and a strengthened glycolysis pathway, which could result in more energy for the generation of ATP and NADPH to resist salt stress [40]. The molecular response of organisms to salt stress may vary depending on species and stress levels.

The early response of *D.salina* to salt stress is a dynamic process. This organism can enhance the tolerance/resistance mechanism and establish cellular metabolic homeostasis under stress conditions. Based on functional and pathway analysis, we revealed the early response mechanisms to acclimatize to salinity in *D.salina*. Firstly, during initial exposure to salinity, changes in photosynthesis proteins may be related to the early response. Secondly, salt stress acclimatization is an energy consuming process. *D.salina* enhances the expression of oxidative phosphorylation-related proteins and the generation of production of ATP repairing of stress-induced damages. Thirdly, salt stress leads to cell instability leading to an increased risk of protein damage. Several heat shock proteins act as molecular chaperones to prevent denaturation and help denature proteins to restore their natural conformation. Furthermore, early stress responses are related to the activity of the protein synthesis system. *D.salina* enhances the processing and renewal of chloroplast and cytoplasmic proteins to cope with salt stress.

A regulatory PPI network was constructed for the DAPs using STRING (Figure 4), which showed considerable interactive networks among proteins involved in photosynthesis, ATP synthesis, and stress responsive signal transduction. Significant interaction was seen between several photosynthesis related proteins including photosystem components (psaA, psaB, psbB, psbC) and ATP synthase subunit proteins (atpA, atpB, atpE). Additionally, ATPC, ATPvL1, atpA, atpB, atpE, psaA, psaB, psbB, psbC, HSP70B, rbl2, and EIF3A, were the most important hubs orchestrating protein regulation in the constructed regulatory network. The downregulation of rbcL, HSP90A, and LHCB5 in the PPI network was consistent with previous findings [9,30,40]. We also observed that LHCB5 was downregulated in *D. salina* after salt treatment. Taken together, our findings indicate that salt stress affected multiple metabolic and physiological pathways in *D. salina*, predominantly photosynthesis, energy metabolism, carbon assimilation and metabolism and heat shock proteins. We also found variations in the DAPs in 3 h versus 24 h response to salt stress, indicating that the alga detected the extent of salt stress and alleviated it by modulating the expression of stress-responsive proteins.

In conclusion, we identified a number of novel proteins whose expression and abundance were significantly altered in the early response to salt stress. Multiple proteins mainly involved in photosynthesis, ATP synthesis, and oxidative phosphorylation, were putatively linked to early *D. salina* salt stress response. Furthermore, important metabolic pathways, including glycolysis, purine, and chlorophyll metabolism were compromised by salt treatment. PPI network analysis suggested that protein metabolism, energy supply, and photosynthesis work together to reconstitute cellular homeostasis under stress. ATPC, ATPvL1, atpA, atpB, atpE, psaA, psaB, psbB, psbC, HSP70B, rbl2, and EIF3A were the most important protein upregulation hubs. The identification of these stress-induced proteins can increase our knowledge of the molecular networks involved in plant salt tolerance, and help to mine more salt stress associated genes. This study provides a better understanding of the molecular mechanisms involved in stress response at the translational level.

## 4. Materials and Methods

### 4.1. Algae Culture

*D. salina* was obtained from the Hydrobiology Laboratory of the Dalian Ocean University (Dalian, China), and maintained at 50 mM photons/m^3^ on alternate 12 h light–dark cycles. The algal cells were cultured in f/2 medium for several weeks in 1 M NaCl as previously described [3,4,41], with temperature and pH maintained at 25 ± 1 °C and 7.5 ± 0.2, respectively. Cells were seeded at a density of ~5 × 10^5^ cells/mL, corresponding to the optical density at 630 nm (OD_630_) of 0.06–0.08. When the cells reached the logarithmic growth phase (~2–3 × 10^6^ cells/mL), they were transferred to fresh medium containing 3 M (salinity shock) NaCl [3,4]. Algal culture with 1 M NaCl addition (normal growth condition) was set as the control. Four replicates were made for the control and salt treatment groups. After 3 or 24 h of 3 M NaCl treatment, algal cells were harvested, and either used fresh or stored at −80 °C for later analyses.

### 4.2. Protein Extraction and Quantification

Proteins were extracted from 2 × 10^8^ cells per sample using Plant Total Protein Lysis Buffer (7 M Urea, 1% CHAPS (3-[(3-cholamidopropyl) dimethylammonio]-1-propanesulfonate), 2 M Thiourea, 40 mM Tris-HCl pH8.5, 2 mM EDTA and 1 mM PMSF) and the cells were sonicated in the buffer for 60 s (0.2 s on, 2 s off, amplitude 25%). Homogenized samples were then incubated for 1 h at 25°C and the remaining debris was removed by centrifugation at 30,000×*g* at 4°C for 30 min. An aliquot of the supernatant was taken and the protein concentration was determined by Bio-Rad DC protein assay (Bio-Rad, Hercules, CA, USA) [42]. A total of 20 μg of protein per sample from cell lysate was subjected to sodium dodecyl sulfate-polyacrylamide gel electrophoresis (SDS-PAGE) to verify protein quality.

### 4.3. Protein Digestionand iTRAQ Labeling and Fractionation by Strong Cationic Exchange (SCX)

For each sample, a total of 200 μg of proteins were precipitated in 4 × volumes of cold acetone overnight at −20 °C. The protein pellets were dissolved in 1% SDS with 100 mM triethylammonium bicarbonate (pH 8.5) and sonicated in ice. Protein samples were reduced and digested with trypsin at 30:1 (*w*/*w*) for 16 h at 37 °C. Peptides were labeled with an iTRAQ Reagents 8-plex kit (AB Sciex Inc., Foster City, CA, USA) and incubated for 2 h at room temperature. The labeled peptide mixtures were then combined and dried by vacuum centrifugation. After labeling, the peptides were reconstituted in solvent A (25% acetonitrile, 25 mM NaH_2_PO_4_, pH 2.7) and then loaded into an Ultremex strong cationic exchange (SCX) column (Phenomenex, Torrence, CA, USA). The peptides were eluted using the SCX column to remove interfering substances such as excess iTRAQ reagents, organic solvents and SDS. The elution process was monitored by measuring the absorbance at 280 nm, and 12 fractions were collected [13].

### 4.4. Liquid Chromatography-Tandem Mass Spectrometry (LC-MS/MS) Analysis

Peptides fraction from each sample were analyzed using a nano-high-performance liquid chromatography (HPLC) system (Shimadzu LC-20AD, Kyoto, Japan) [25]. The 100-μL labeled peptides were resolved in solvent A containing 5% acetonitrile and 0.1% formic acid. Samples with individual volumes of 10 μL were loaded into a C18 trap column. Subsequently, solvent B (95% acetonitrile *v*/*v*, 0.1% formic acid) was used to separate the peptide with the following linear gradient conditions. Peptides were eluted with a flow rate of 0.6 mL/min. The elution peptide gradient was used from 5% solvent B to 35% solvent B for 35 min, then ramped up to 60% solvent B over five minutes, raised to 80% in two minutes and held for five minutes. The LC elute was then subjected to a Q Exactive MS (Thermo Fisher, NY, USA) coupled online to the HPLC. The applied electrospray voltage was 2.5 kV.

### 4.5. Analysis of Differentially Abundant Proteins

Protein identification and quantification was performed using the Mascot 2.3.02 search engine (Matrix Science, Boston, MA) against the UniProt database (http://www.uniprot.org). Viridiplantae (39,754 entries in UniProt) was chosen for taxonomic categorization. All DAPs were compared to the *D.salina* genome database (http://genomeportal.jgi.doe.gov) to further identify the annotated protein entries [43]. The protein mass were predicted using online software (http://www.expasy.ch/tools/) on the basis of the protein sequences. The peptide mass tolerance was set as ±20 ppm and the fragment mass tolerance was 0.1 Da. The results were filtered based on a false discovery rate (FDR) of no more than 0.01. To demonstrate the reproducibility of the replicates, protein abundances between various biological replicates were compared, and ratios for each protein comparison were normalized to 1. Only proteins with at least one unique peptide and unused value of >1.2 were considered for further analysis. When differences in protein expression between salt-treated and control groups were >1.2-fold or <0.83-fold [22,23], with a *p*-value < 0.05, the protein was considered to be differentially abundant. The mass spectrometry proteomics data have been deposited to the ProteomeXchange Consortium via the PRIDE [44] partner repository with the dataset identifier PXD010739.

### 4.6. Go, KEGG and STRING Enrichment Analyses

Functional analysis of DAPs was based on biological process, molecular function, and cellular components, using GO annotation and protein classification (http://www.geneontology.org) [45]. The DAPs were further assigned to the KEGG database (http://www.genome.jp/kegg) and the STRING database (http://www.string-db.org/). KEGG was used to predict the major metabolic and signal transduction pathways involved in the identified DAPs [46,47]. STRING was used for protein interaction analysis in order to identify protein interaction networks of *D.salina* under salinity stress conditions. The protein interaction (PPI) networks’ responses to salinity stress were obtained [48].

### 4.7. RNA Extraction and qRT-PCR

Total RNA was extracted from *D. salina* using Trizol (Invitrogen, Carlsbad, CA, USA) according to the manufacturer’s instructions. The RNA quality was analyzed with a NanoDrop 2000 spectrophotometer (Thermo, USA), after which cDNA was synthesized using the PrimeScript Reverse Transcriptase Kit (Takara, Japan), and cDNAs were amplified and detected using SYBR Green PCR Kit (Qiagen, Valencia, CA, USA). qRT-PCR was completed using the ABI 7500 Real-Time PCR system (Applied Biosystems, Foster City, CA, USA). The 18s-rRNA gene served as an endogenous control for normalization. The details of gene-specific qRT-PCR primers are listed in Supplementary Table S4. The primers were designed using the Primer-BLAST program (https://www.ncbi.nlm.nih.gov/tools/primer-blast) based on National Center for Biotechnology Information (NCBI) sequence data (http://www.ncbi.nlm.nih.gov/genbank/). The relative expression level was calculated as follows: ratio = 2^−ΔΔ*C*t^ = 2^−(Δ*C*tt–Δ*C*tc)^ (Ct: cycle threshold; Ctt: Ct of the target gene; Ctc: Ct of the control gene) [49].

## Supplementary Materials

Supplementary materials can be found at http://www.mdpi.com/1422-0067/20/3/599/s1.

## Abbreviations

iTRAQ	Isobaric Tags for Relative and Absolute Quantitation
DAPs	differentially abundant proteins
BP	biological process
MF	molecular function
CC	cellular components
GO	Gene Ontology
KEGG	The Kyoto Encyclopedia of Genes and Genomes
STRING	Search Tool for the Retrieval of Interacting Genes
PPI	predict protein interaction
LHC	light-harvesting complex
qRT-PCR	quantitative real-time polymerase chain reaction
SDS-PAGE	sodium dodecyl sulfate -polyacrylamide gel electrophoresis
SCX	strong cationic exchange
LC-MS/MS	liquid chromatography-tandem mass spectrometry
